# Mycosynthesis of chitosan-selenium nanocomposite and its activity as an insecticide against the cotton leafworm *Spodoptera littoralis*

**DOI:** 10.1038/s41598-024-81988-6

**Published:** 2025-01-06

**Authors:** Sohila A. Ibrahiem, Fifi M. Reda, Eman M. Abd-ElAzeem, Mostafa S. Hashem, Hala A. Ammar

**Affiliations:** 1 Plant Protection Research Institute, Agriculture Research Center, Giza, Egypt; 2 Botany and Microbiology Department, Faculty of Science, Zagazig University, Zagazig, Egypt

**Keywords:** *Spodoptra littoralis*, Selenium nanoparticles, Chitosan selenium nanocomposite, *Penicillium griseofulvum*, Entomology, Nanofabrication and nanopatterning, Microbiology

## Abstract

The cotton leafworm, *Spodoptra littoralis,* causes great damage to cotton crops. A new, safer method than insecticide is necessary for its control. Selenium nanoparticles (SeNPs) are metalloid nanomaterial, with extensive biological activities. They have low toxicity and can be used safely in plant disease management. In this study, we successfully bio-fabricated selenium nanoparticles and chitosan-selenium nanocomposite (Ch-SeNPs) using a fungal cell-free filtrate of *Penicillium griseofulvum*. The biosynthesized nanomaterials were initially detected optically by the formation of a red color in the solution mixture and the appearance of a strong plasmon resonance peak at 240–300 nm. The biosynthesized nanomaterials were fully characterized by UV–visible spectroscopy, transmission electron microscopy, dynamic light scattering, energy dispersive X-ray, inductively coupled plasma spectroscopy, and Fourier transform infrared. We tested the anti-insect activities of SeNPs, and Ch-SeNPs against larvae of *S. littoralis* compared to spore suspensions of *P. griseofulvum*. The results indicated that Ch-SeNPs followed by SeNPs gave a significantly higher mortality percentage than the spore suspension of the tested fungus. The highest production of all biosynthesized nanomaterials was detected after 7 days at 40 °C under alkaline conditions (pH 9). The average size diameter of SeNPs and Ch-SeNPs were 91.25 and 67.41 nm with zeta potential − 8.05 and + 41 mV, respectively. Both Ch-SeNPs and SeNPs gave high mortality rates and low values of LC_50_ and LC_90_ for both larvae and pupae. Ch-SeNPs showed stronger activity against *S. littoralis* than SeNPs and spore suspension at all experimental conditions. Cytotoxicity experiments indicated their safety against honeybee populations. The current study reveals the significant ultrastructure impact of SeNPs on larvae. These findings suggest that selenium nanoparticles and nanocomposite can be fabricated with a costless easy route using fungal filtrate, and they can be used safely in pest control systems that are safe for honeybee populations. It is the first report about the application of Ch-SeNPs as an anti-insect agent.

## Introduction

Potatoes are a relatively recent introduction to Egypt’s agricultural landscape, and their consumption might have increased due to their nutritional value and versatility in cooking. Potatoes are a good source of carbohydrates, vitamins, and minerals, making them an important food source in many parts of the world^1^. *S. littoralis* can cause significant damage to different crops, including potatoes. This pest is a member of the Lepidoptera order and is considered a polyphagous insect. The larvae of *S. littoralis* are voracious feeders. They consume the leaves of potato plants leading to defoliation and a reduction in yields^2^. *S. littoralis* has developed resistance to many chemical insecticides over time. Because traditional chemical treatments may not be effective in controlling its population, pest management is more challenging^3^. Controlling *S. littoralis* infestations in potato crops requires an integrated pest management approach, including a combination of biological control methods^4^.

Fungi are commonly used as biological control agents in the management of various pests and plant diseases. This biocontrol approach, using naturally occurring or specifically cultivated fungi, was used to reduce the populations of harmful pests. Fungi can have both direct and indirect effects on pests and pathogenic populations, making them valuable tools in integrated pest management strategies^5^. In recent years, selenium nanoparticles (SeNPs) have gained attention for their potential applications in various fields, including agriculture and pest management^6^. Selenium is an essential trace element for many organisms, and its nanoparticles have shown antimicrobial and insecticidal properties. Some research explored the potential of selenium nanoparticles as a tool for pest management^7^.

Selenium nanoparticles have been studied for their insecticidal effects on various insect pests. It is believed that these nanoparticles could disrupt insect physiology, such as by damaging their cuticles, affecting their respiration, or interfering with their development. Also, selenium is a naturally occurring element and is generally considered less harmful to the environment compared to some chemical pesticides. This makes selenium nanoparticles an interesting candidate for eco-friendly pest control methods. Selenium is a naturally occurring element and is generally considered less harmful to the environment compared to some chemical pesticides. This makes selenium nanoparticles an interesting candidate for eco-friendly pest control methods^8^. On the other hand, chitosan is a biocompatible and biodegradable polymer considered safe for human use. Several studies have mentioned the use of chitosan as a polymeric nanoparticle carrier in drug delivery systems^9^. Nakashima et al.^10^ recorded that Se/Ch-nanoconjugate may be more easily absorbed by tumor cell lines and have the most anticancer activity. We aimed in this study to investigate an eco-friendly route for pest management control of the cotton leafworm *S. littoralis* using the bio-fabricated SeNPs and Ch-SeNPs in comparison with fungal spore suspension of *P. griseofulvum*.

## Methods

### Chemicals

Selenium dioxide (SeO_2_), potato dextrose broth medium (PD broth), Chitosan M.W (100.000–300.000), and all other chemicals used in this study were purchased from Sigma–Aldrich company (St Louis, MO, USA).

### Rearing technique of *S. littoralis*

The laboratory strain of *S. littoralis* used in this study was obtained from a laboratory of the leaf worm Research Department, Plant Protection Research Institute, Sharkia branch, Egypt. This colony was reared for several generations away from any contamination with insecticides on castor bean leaves*, Ricinus communis* L. (as a source of food that was provided daily until pupation). It was incubated under controlled conditions, at 26 ± 1 °C and 70 ± 5% RH, as described by El-Defrawi^11^. Pupae were transferred individually to other clean tubes and incubated at the same previous conditions until the month’s emergence. The adults were sexed and placed in glass jars (500 mL volume) supplied with leaves of Tafla (*Nerium oleander* L.) for egg-laying. Adults were fed a 10% sugar solution and changed to a new one daily. The obtained eggs were kept in glass jars (500 mL volume) and incubated at the same conditions until hatching. The newly hatched larvae were used directly in the experiments.

### Isolation and identification of fungal species

Twenty-five fungal strains were isolated from different Egyptian soils. The fungal filtrates of all isolates were tested for the biosynthesis of selenium nanoparticles. Five species were selected as potential producers of SeNPs. *P. griseofulvum* was selected as an experimental organism and exposed for morphological and molecular identification. Morphological identification was proceeded by microscopic examination on PDA medium for 5 days incubated at 30 °C, and it was identified to genus and species level using the taxonomic key proposed according to Pitt et al.^12^.

The morphological identification of *P. griseofulvum* was confirmed by the molecular identification of rRNA, based on the genetic markers ITS1 and ITS2, using ITS1/ITS4 primers. The universal sequences of the ITS1 and ITS4 primers were 5′-TCCGTAGGTGAACCTGCGG-3′ and 5′TCCTCCGCTTATTGATATGC-3′, respectively^13^. The obtained sequence of *P. griseofulvum* was registered with NCBI GenBank under accession number OR672743. Thereafter, it was analyzed by the BLAST program to detect its ratio of similarity with the other related sequences deposited in the GenBank database. Molecular evolutionary genetic analysis (MEGA version 11) software was used for phylogenetic analysis^14^. A phylogenetic tree was constructed using the maximum composite UPGMA method with 1000 bootstrap replicates based on ITS gene sequences. The closet homologous sequences were selected, and multiple sequence alignment was carried out using the ClustalW program in the MEGA11 software. Evolutionary history was inferred using the UPGMA method.

### Biosynthesis of selenium nanoparticles

#### Production of fungal biomass

Spore suspensions of each fungal isolate (1 × 10^7^ spores mL^−1^) were inoculated aerobically in 250 mL Erlenmeyer flasks containing 50 mL of PD broth. The culture media was incubated at 30 °C for 7 days under shaking conditions at 150 rpm. The fungal biomass was harvested and washed three times with sterilized bi-distilled water using filter paper Whatman No. 1 to remove any traces of medium. 10 g of wet biomass were suspended in 50 mL of sterilized bi-distilled water, then incubated at 30 °C for 3 days under shaking condition at 150 rpm. The fungal suspension was separated by Whatman filter paper No. 1, and fungal cell free filtrate (CFF) was used for biosynthesis of SeNPs and Ch-SeNPs.

#### Biosynthesis and optimization of SeNPs and Ch-SeNPs

An equal volume of each fungal CFF was mixed with 3 mM SeO_2_. The reaction mixture was incubated in statically in a dark place at 40 °C for 72 h. The biosynthesizing conditions were optimized for obtaining the highest production of nanoparticles after a short time. The solution mixture was incubated at different temperatures (40 and 60 °C), different pH values (3, 7, 5 and 9) and various incubated time (1, 3, 7, 14, 21 and 30) days.

For biosynthesis of Ch-SeNPs, an equal volume of 3 mM SeO_2_ was mixed with fungal filtrate of *P. griseofulvum* in flasks. 0.5 mL of 1% of polymer (chitosan) was added, separately, drop by drop into the previous mixtures. The mixture was kept for magnetic stirring at 40 °C for 1 h, then all mixtures (sample and control) were incubated at optimum conditions and checked for Ch-SeNPs formation by UV–visible spectrophotometer, DLS, TEM, FTIR and Zeta potential.

### Characterization of nanoparticles

#### Visual observation

The prepared nanoparticles were first characterized by a visual observation of color change for the nanoparticle solution with the formation of red-yellowish color due to the reduction of selenium ions. The bio-reduction of selenium ions was estimated by UV–visible spectrophotometer. Absorption measurements were carried out at a resolution of 1 nm and the absorption spectrum of colloidal solution of nanoparticles was scanned in the range of 200–800 nm.

#### Dynamic light scattering (DLS)

Dynamic light scattering system (DLS) was used for evaluation of hydrodynamic diameter and distribution of particles in the solution. The solid particles distribution of Selenium nanoparticles in the liquid solution was determined by Zeta sizer Nano ZS ZEN3600, Malvern Instruments Ltd (Worcestershire, UK). Measurement of Zeta potential was carried out by the same instrument. Estimation of Zeta potential indicates a concept about the charges and the stability of nanoparticles, and its determination based on laser Doppler electrophoresis technique in the same instrument.

### Transmission electron microscopy (TEM)

The shape, dispersity, and size of both SeNPs and Ch-SeNPs was determined by TEM (JEOL-2100 Japan). 2 μL of nano-solution was loaded on a formvar-coated 200-mesh copper grid (Ted Pella, Redding, CA, USA), dried in air, and then loaded onto a specimen holder. TEM micrographs were taken and then the shape and size of nanoparticles were evaluated, their mean diameter was determined with Image J software.

#### Fourier transform infra-red spectroscopy (FTIR)

The principal functional groups formed on the surface of nanoparticles were estimated by Fourier transform infrared spectroscopy (FTIR). The measurements were carried out using FTIR spectroscopy (JASCO, FT/IR- 6100), by employing the KBr pellet technique. The spectra were scanned in the range of 400–4000 cm^−1^ at a resolution of 4 cm^−1^.

#### Energy dispersive X-ray analysis (EDX)

Energy dispersive X-ray analysis (EDX) is used to detect the elemental details of the nanoparticle samples. This technique is performed in conjunction with scanning electron microscope (SEM). A high-energy electron beam is bombarded on the nanoparticles samples and X-rays emitted from the sample are collected by an energy dispersive spectrometer.

#### Inductively coupled plasma spectroscopic analysis (ICP)

The elemental metal concentrations of SeNPs were determined by inductively coupled plasma spectroscopy (ICP) (Ultima 2 JY optical emission spectrophotometer, France). Soluble selenium in aqueous solution samples was stored by addition of 1 mL Nitric acid for 100 mL solution and filtered through filter paper, Whatman No. 42. The concentration of selenium was calculated as mg^−l^ and compared with the original salt concentration in the solution.

#### Optimization of nanoparticles production

The solution mixture was adjusted for different pH values (3, 5, 7 and 9) and incubated at different temperatures (40, and 60 °C) in the dark condition for different incubation time (1, 3, 7, 14, 21 and 30 days). The pH values were measured by pH meter (AD1030).

#### Biological activity of biosynthesized nanoparticles against *S. littoralis*

To study the biological effects of the nanoparticles on the 2nd instar larvae of *S. littoralis*, leaves of castor bean were dipped for 10 s in the nanoparticles solutions and left to dry, then placed in glass gars (500 mL volume). Twenty-five 2nd instar larvae of *S. littoralis* were added to each glass gar. Four replicates were used for each treatment and control. Leaves dipped in distilled water were used as a control. Glass gars were covered by pieces of cloth and incubated at previous conditions. Larvae were allowed to feed on treated leaves for 24 h, then provided with untreated clean castor leaves. The larvae were examined daily to record the biological parameter, larval duration, larval mortality, and pupation percentage. The pupae were transferred to clean gars and incubated until moth emergence. Pupal duration and pupal mortality percentages were recorded. The emerged adults were sexed and placed (3 pairs/replicate) in a glass gar (500 mL volume) supplied with Tafla leaves for laying eggs. Adults were fed on a 10% sugar solution and changed by new one daily. Four replicates were used for each treatment and control.

#### Toxic effect of SeNPs and Ch-SeNPs on the treated larvae

To evaluate the toxic effect SeNPs and Ch-SeNPs against 2nd larvae, 1 mL containing different concentrations of each nano-selenium, 50, 100 150 and 200 ppm, was distributed on the surface of artificial diet. 1 mL of water and 1 mL of fungal metabolite were used separately as a control. Twenty-five larvae of the *S. littoralis* were transferred by a fine brush to the surface of four replicates of each treated diet in addition to the control. The larvae were incubated for 24 h at 27 °C, then the larval mortality was recorded. The LC50 and LC90 values were calculated according to the method described by Finney^15^.

Toxicity index (T.I) and relative toxicity were determined according to Sun^16^ as follows:

$${\text{Toxicity index }}\left( {{\text{T}}.{\text{I}}.} \right) = \frac{{{\text{LC}}50{\text{ of the compound }}\left( {\text{A}} \right)}}{{{\text{LC}}50{\text{ of the compound }}\left( {\text{B}} \right)}}{ } \times 100$$

where A is the most effective compound, B is the other tested compound.

The relative potency (R.P.) values were measured according to Zidan and Abdel-Megeed^17^.

$$\text{Relative potency }(\text{R}.\text{P}.)\hspace{0.17em}=\hspace{0.17em}\frac{\text{LC}50\text{ of the lowest toxic insectcide}}{\text{LC}50\text{ of the lowest tested insectcide}}$$

#### Biochemical profiling of *S. littoralis* larvae after treatment with selenium nanoparticles

The present experiment has been designed to study the changes in the activities of carbohydrate enzymes (amylase, Invertase and Trehalase), acetylcholine esterase (AChE) and total soluble protein in supernatant of homogenate 2nd larvae after treatment with nanoparticles solution compared to untreated larvae (control).

#### Preparation of samples

After 24 h of treatment, 20 larvae from each treatment in addition to control sample were transferred into clean jars and left to starve for 4 h. The starved larvae were homogenized in distilled water (1 g larva/mL) using a Teflon homogenizer surrounded with jacket of crushed ice for 3 min. The homogenate larvae were centrifuged at 3500 rpm for 10 min at 5 °C. The supernatant was immediately assayed to determine the enzymes activity.

#### Estimation of enzymes activity

The activity of amylase, invertase and trehalase enzymes were estimated according to Ishaaya and Swiriski^18^. The optical density (O.D.) of the produced color was measured at 550 nm using a spectrophotometer. The enzymatic activity was expressed as mg glucose released/g body weight/min. Colorimetric determination of total soluble protein in total homogenate of larvae was carried out according to Gornall et al.^19^. The larval homogenate (0.2 mL) was added to 5 mL of Biuret reagent and incubated for 30 min at 20–25 °C. The absorbance of the sample against a blank Biuret reagent was measured at wavelength of 546 nm.

#### Transmission electron microscopy (TEM) of treated larva

TEM analysis was used to investigate the effect of Ch-SeNPs on the mid gut of the untreated and treated 2nd instar larva after 7 days.

#### Cytotoxic activity of selenium nanoparticles

Honeybees *Apis mellifera* were selected as a model insect to study the cytotoxic activity of SeNPs and Ch-SeNPs against beneficial insects. Worker bees were collected from the peripheral combs of the colony. The worker bees, used in this study were collected from one colony headed by open mated F1 Carniolan queen, educational and research apiary of Plant Protection Institute, Egypt. The tested worker bees were slightly anaesthetized before their treatments with nanoparticle solution. To investigate the effect of nanoparticles against honeybees, SeNPs and Ch-SeNPs were mixed separately with the feeds of bees. The nanoparticle’s solution was added to sucrose syrup and fed to the bees in feeding cages (9 × 12 × 20 cm). The worker bees were fed on sucrose syrup only as a control. The experiment was carried out at room temperature and 52–60% relative humidity. The numbers of dead bees were counted after 24 and 48 h of feeding. The mortality percentages were evaluated according to the Abbott formula.

### Statistical analysis

All data were statistically analyzed according to completely randomized design. The appropriate methods were used for the analysis of data according to^20^ and the proper “F” value was calculated as described by Fisher and Hills^21^ and Snedecor^22^.

## Results

### Identification of fungal strains

**Fig. 1:**
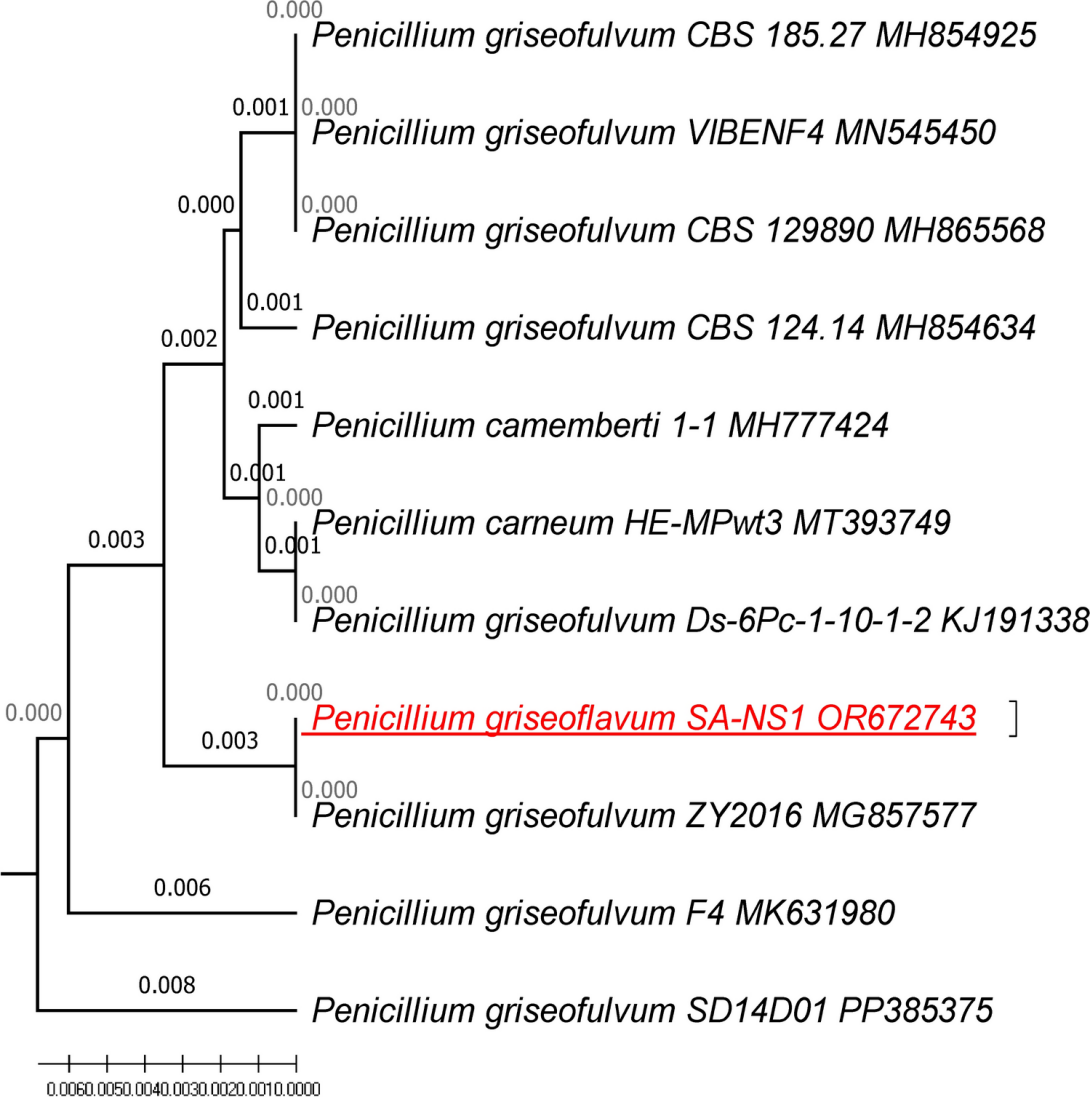
Phylogenetic analysis of *P. griseofulvum* SA-NS1 (OR672743) showing its relationship with the most related species deposited in the GenBank database. Evolutionary history was inferred using the UPGMA method. The tree is drawn to scale, with branch lengths (above the branches) in the same units as those of the evolutionary distances used to infer the phylogenetic tree. The evolutionary distances were computed using the Maximum Composite Likelihood method and are in the units of the number of base substitutions per site. This analysis involved 11 nucleotide sequences. All ambiguous positions were removed for each sequence pair (pairwise deletion option). There was a total of 605 positions in the final dataset. Evolutionary analyses were conducted in MEGA11 software.

*P. griseofulvum* was selected in this study as the most potential strain for production of SeNPs and the highest mortality percentage against larvae of *S. littoralis*. The morphological identification of *P. griseofulvum* was confirmed by molecular methods, based on the genetic marker18S–28S rRNA sequence. The retrieved sequence was deposited in the GenBank under the accession number OR672743*.* Figure 1 showed that the fungal strain represents 100% similarity with the closely related fungi submitted in the GenBank database.

#### Biosynthesis and optimization of SeNPs

**Fig. 2:**
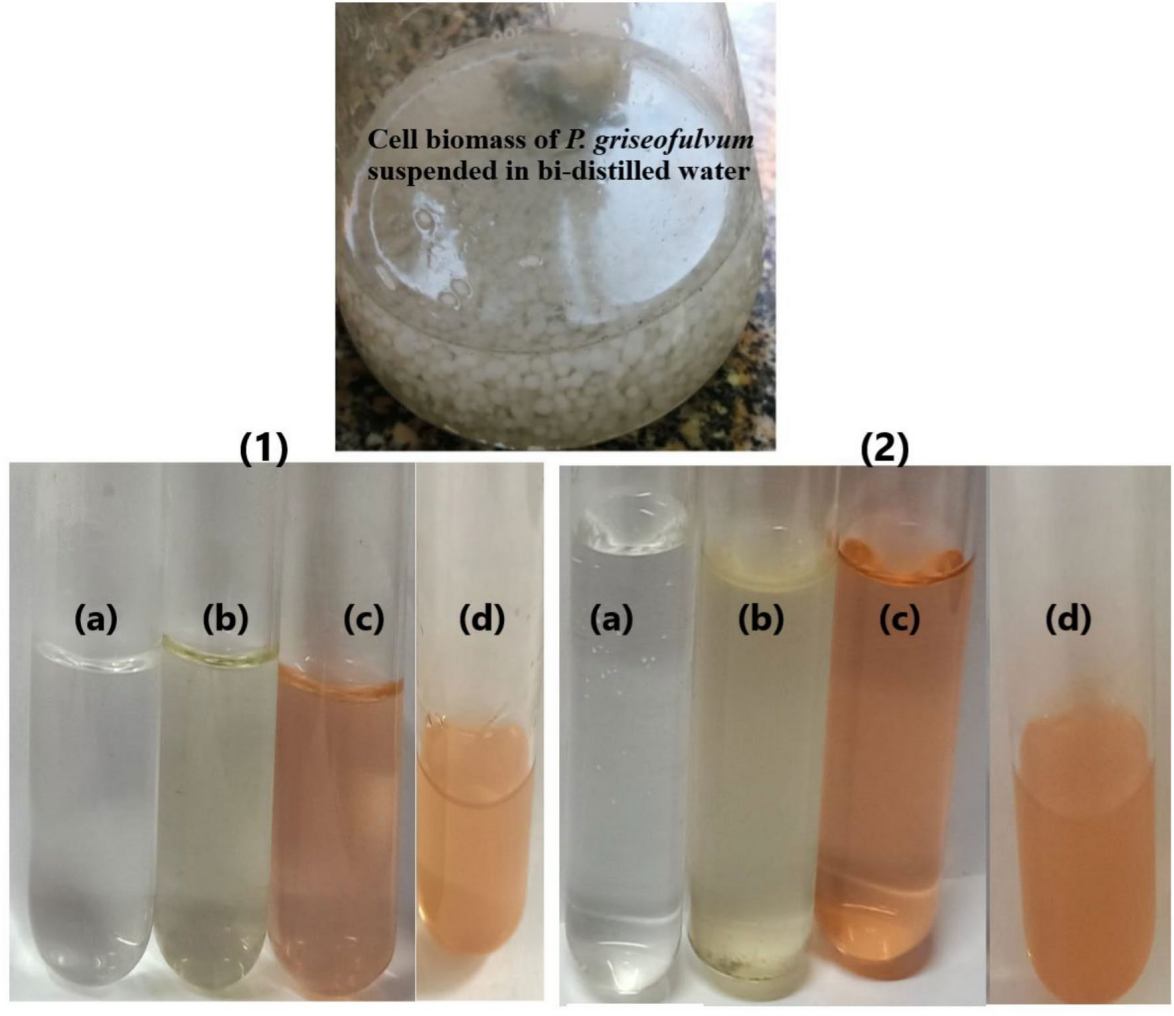
Mycosynthesis of selenium and chitosan selenium nanoparticles. Selenium salt (**a**), fungal filtrate (**b**), SeNPs (**c**), Ch-SeNPs (**d**) after 7 days of incubation at 40 °C (1) and 60 °C (2).

**Fig. 3:**
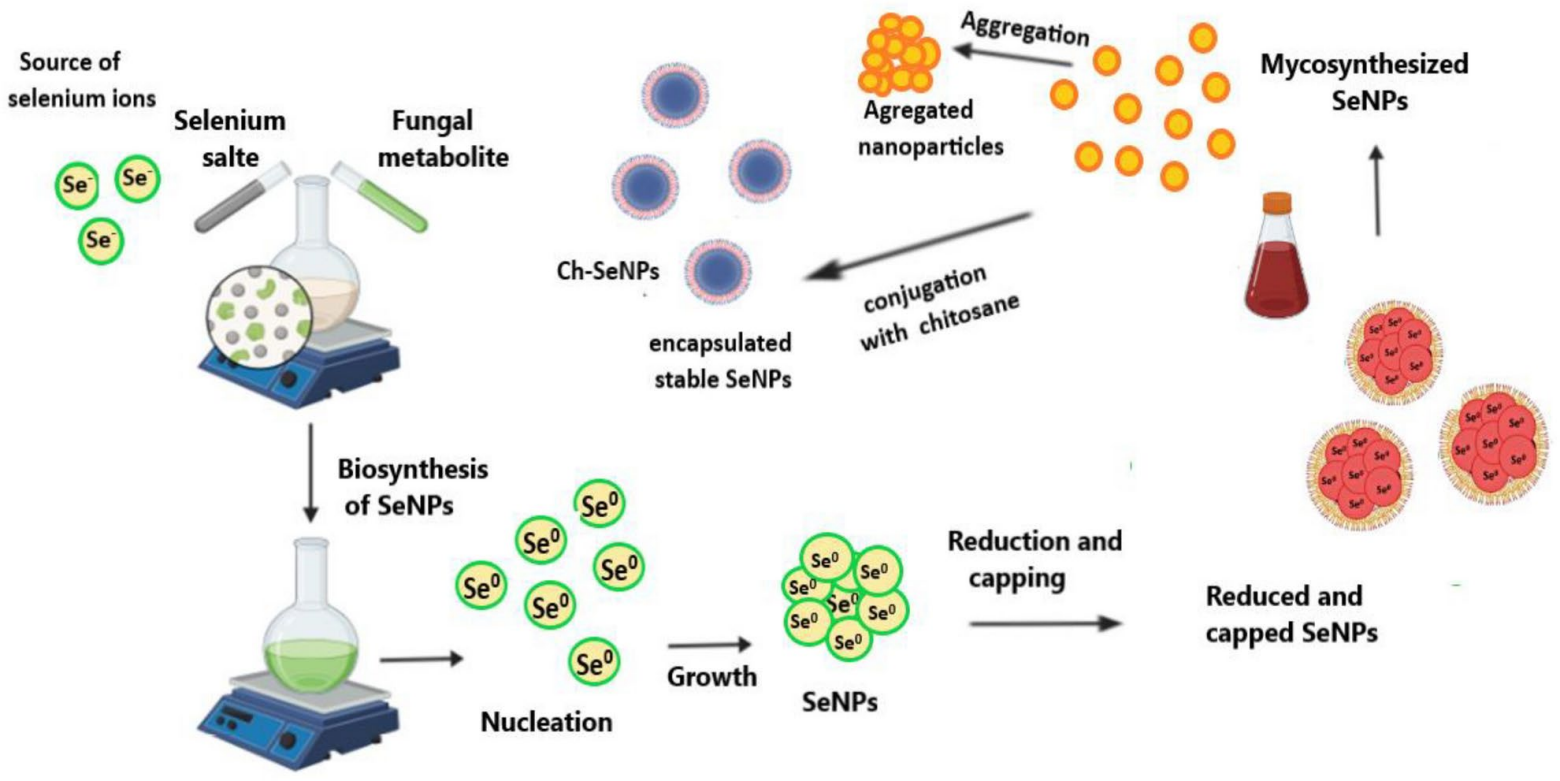
Diagrammatic illustration of the mechanism of the biosynthesizing process of SeNPs and Ch-SeNPs using fungal metabolite**.**

**Fig. 4:**
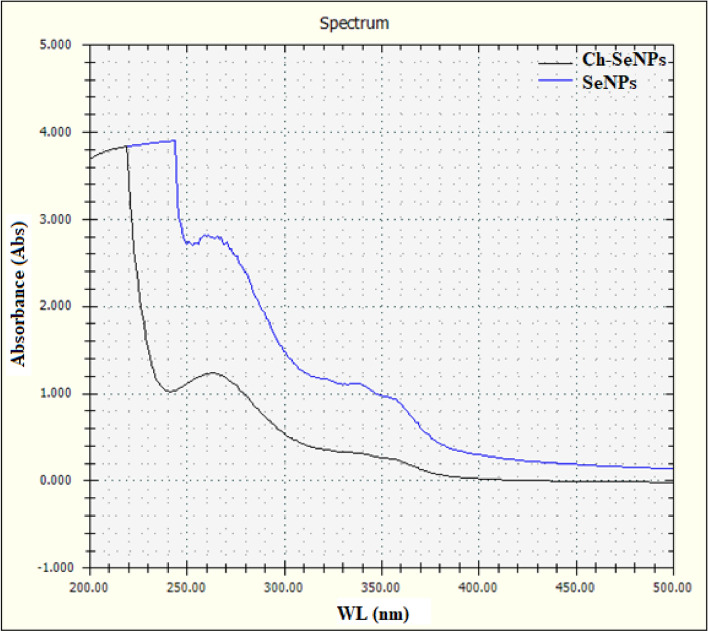
UV spectroscopic analysis of SeNPs and Ch-SeNPs.

The color of fungal filtrate was changed to yellowish red after its treatment with selenium salt. The same changes in the solution mixture were detected after the addition of chitosan and incubation for 7 days at 40 °C which indicated the formation of Ch-SeNPs (Fig. 2). The mechanism of biosynthesizing process was summarized in Fig. 3. Both SeNPs and Ch-SeNPs were screened by UV-visual spectrophotometer at the range of 200–800 nm. It was observed that the maximum absorbance peaks of SeNPs and Ch-SeNPs appeared at 265 and 352 nm, respectively (Fig. 4). Optimization conditions of the reaction mixture indicated that the optimum production of nanoparticles was detected at 9 PH value after 7 days of incubation at 40 °C, whereas the optimum formation of Ch-SeNPS was detected at pH 3 after 7 days of incubation at 40 °C (Tables 1 and 2).

**Table 1:** Effect of different PH values on biosynthesis of SeNPs and Ch–SeNPs .

pH value	3	5	7	9	*P*	F test	LSD_0.05_
Ch-SeNPs							
Wavelength	338d	343c	349b	352a	0.001	***	1.883
Absorbance intensity	1.622a	1.556b	1.413c	1.38d	0.001	***	0.0019
SeNPs							
Wavelength	248d	256c	260b	270a	0.001	***	1.883
Absorbance intensity	1.125	1.228	1.188	1.301	0.001	***	0.0019

The mean value followed by different letters (a, b, c, d, e) within the same row is significantly different (One-way ANOVA, *P* ≤ 0.05).

LSD: the least significant difference.

***Means highly significant.

**Table 2:** Effect of different temperatures for different incubation periods on Se NPs biosynthesis.

Temperatures	Days	Ch-SeNPs		SeNPs	
		Wavelength	Absorbance intensity	Wavelength	Absorbance intensity
40	1	330d	1.102g	261	1.007h
40	3	328e	1.523b	260	1.398d
40	7	352a	1.617a	265	1.841a
40	14	351ab	1.381c	262	1.509b
40	21	326f	1.201f	263	1.190f
40	30	338c	1.243e	261	1.196e
60	1	350b	1.301d	262	1.404c
60	3	350b	1.301d	262	1.404c
60	7	337c	1.020h	260	1.071g
*P value*		0.001	0.001	0.002	0.001
F-test		***	***	***	***
LSD _0.05_		1.715	0.0017	1.715	0.0017

The mean value followed by different letters (a, b, c, d, e) within the same column are significantly different (One-way ANOVA, *P* ≤ *0.05*).

LSD: the least significant difference.

***Means highly significant.

#### Characterization of SeNPs and Ch-SeNPs

**Fig. 5:**
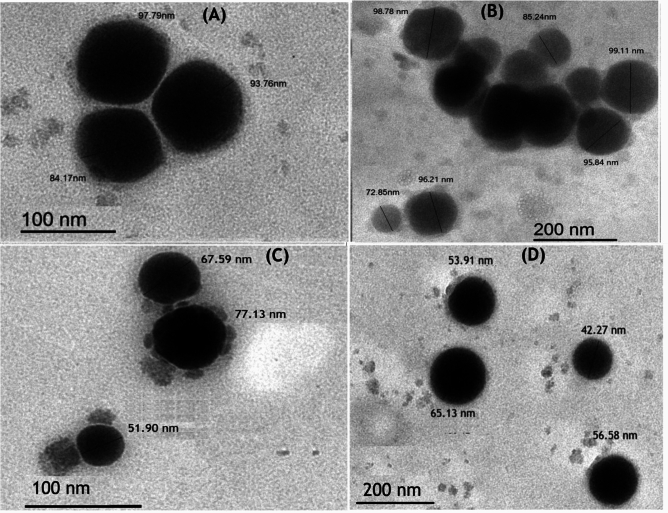
TEM image of SeNPs (**A** and **B**) and Ch-SeNPs (**C** and **D**) from two different magnifications.

**Fig. 6:**
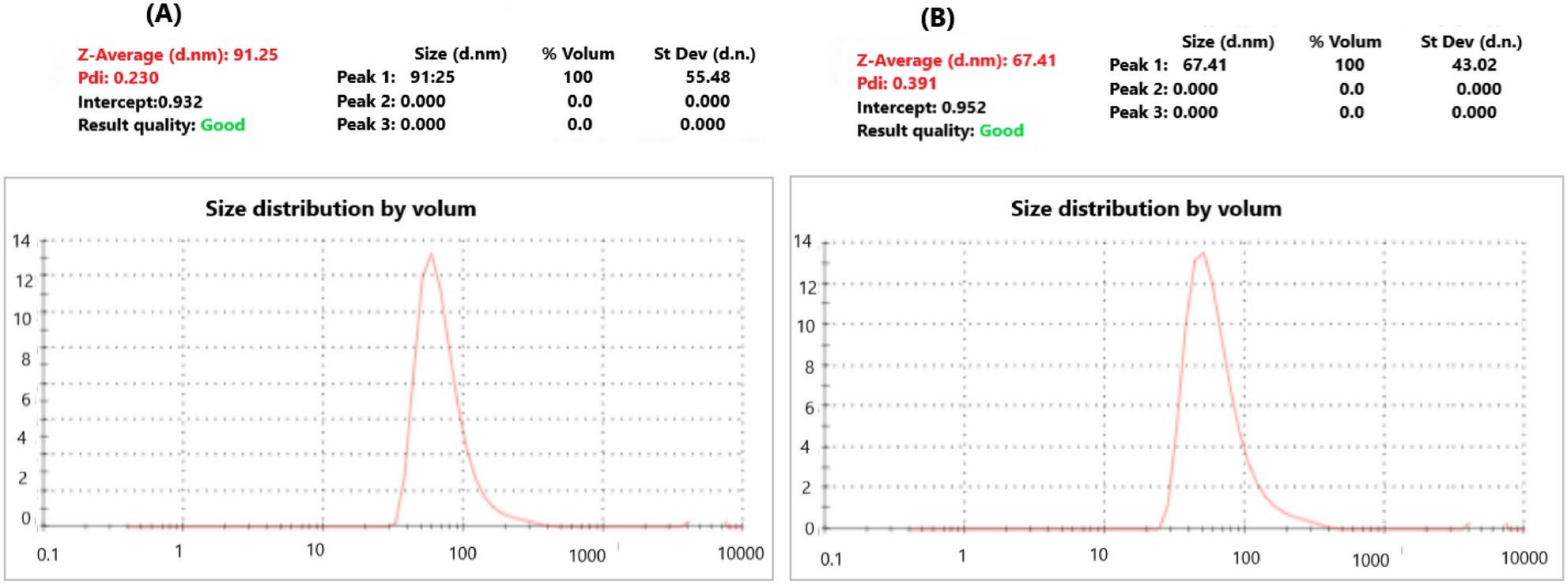
Zeta sizer analysis of SeNPs (**A**) and Ch-SeNPs (**B**) showing size distribution of nanoparticles in the solution mixture.

**Fig. 7:**
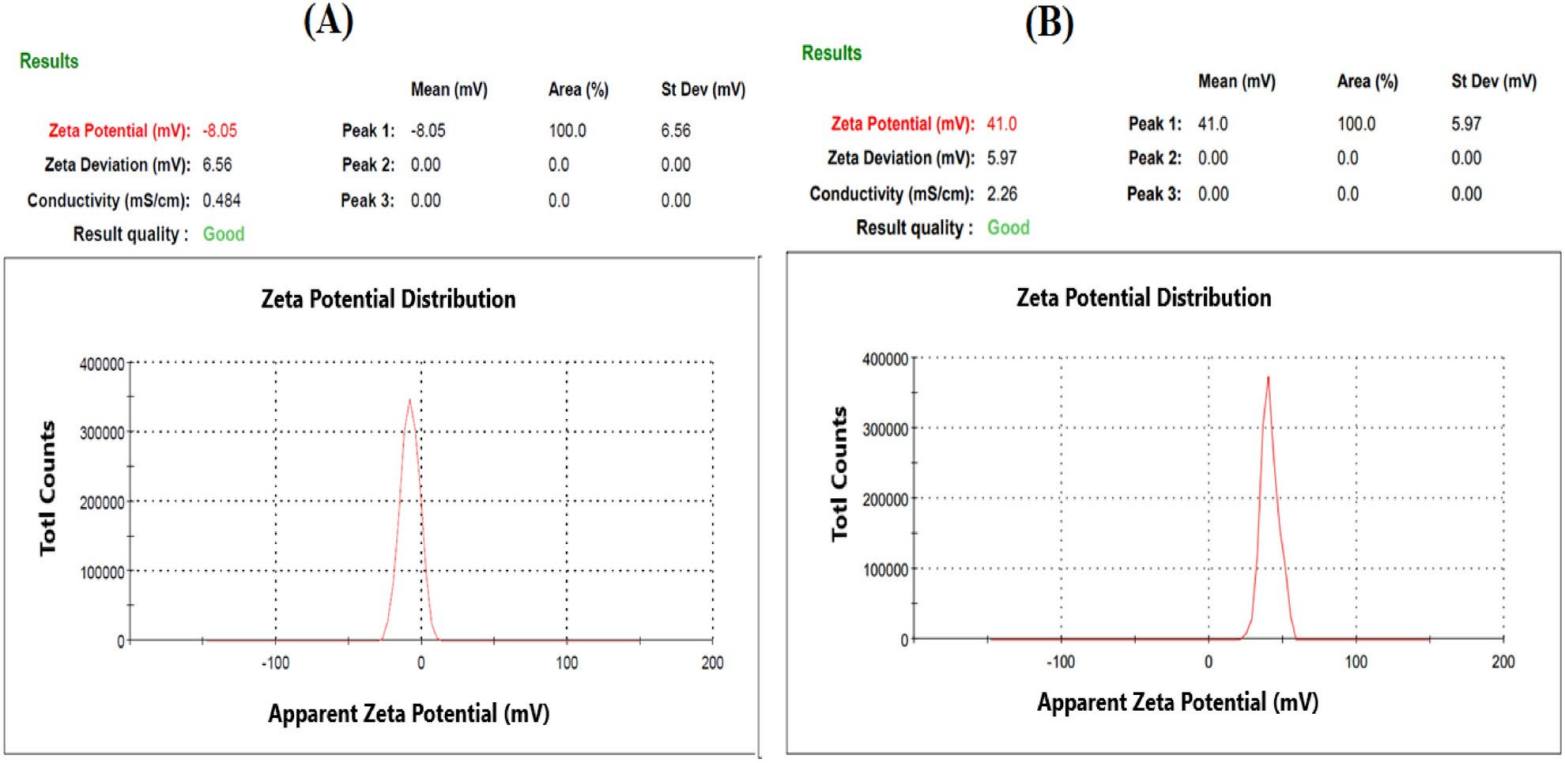
Zeta potential of SeNPs (**A**) and Ch-SeNPs (**B**) showing the charge distribution on the particles surface.

**Fig. 8:**
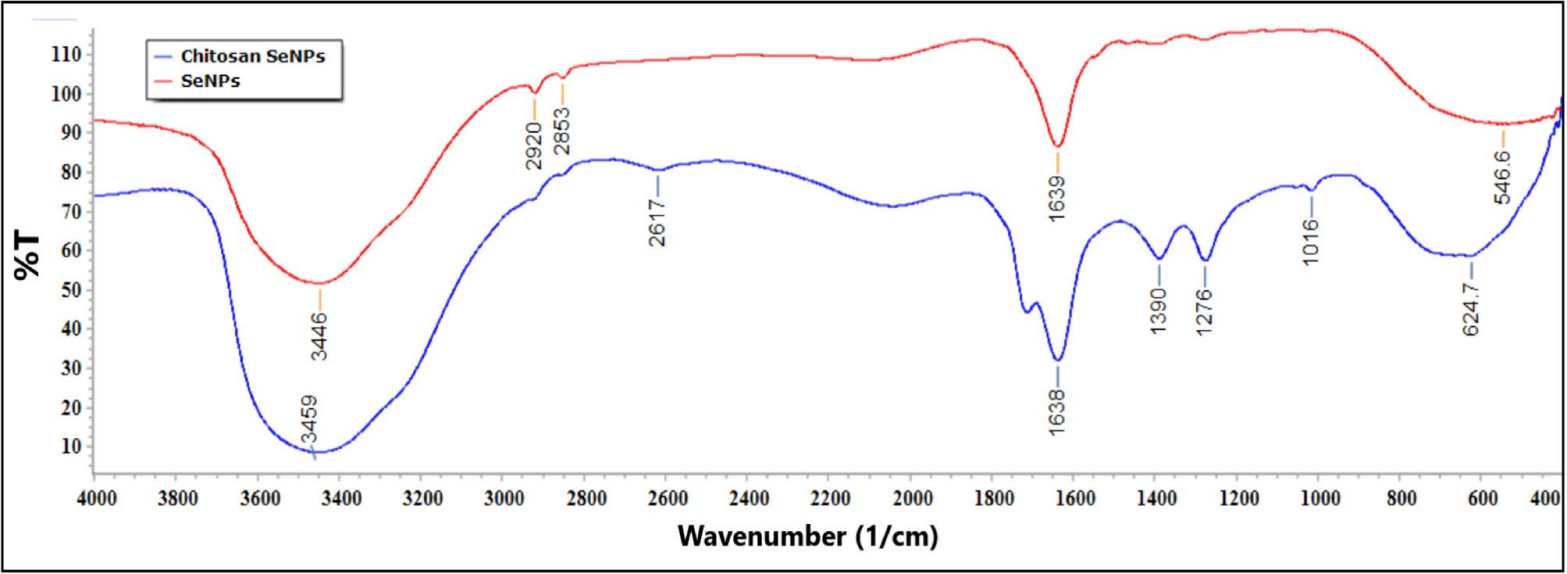
Fourier transform infrared spectroscopy (FTIR) analysis of SeNPs and Ch-SeNPs.

**Fig. 9:**
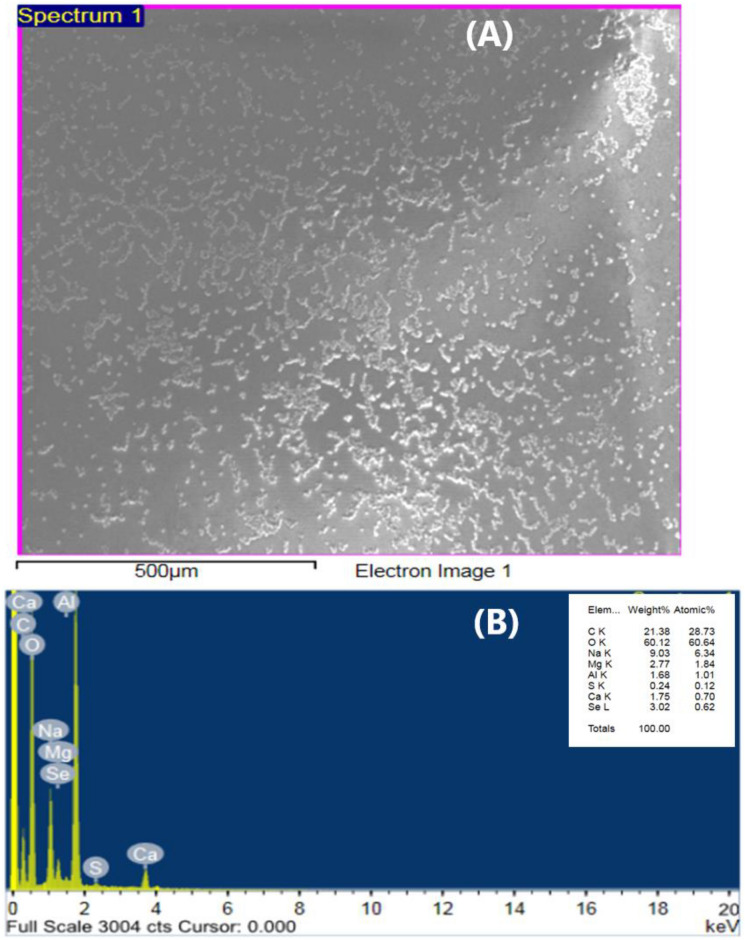
SEM analysis (**A**) and Energy dispersive X-ray (EDX) spectrums (**B**) of selenium nanoparticles.

The biosynthesized nanoparticles were identified by DLS, TEM, and zeta potential analyses. The morphological structure of SeNPs was imaged by TEM as shown in Fig. 5. Well dispersed spherical nanosized particles of both SeNPs and Ch-SeNPs appeared in the TEM images. Size distribution analysis of the biosynthesized nanoparticles was performed using dynamic light scattering (DLS) in aqueous solution (Fig. 6). The average size diameter of both SeNPs and Ch-SeNPs were 91.25 and 67.41 nm, respectively. Zeta potential analysis of SeNPs was − 8.05 and changed to + 41 after their binding with chitosan (Ch-SeNPs) as shown in Fig. 7. FTIR analysis was carried out to identify the types of chemical group responsible for the nanoparticle’s formation. Strong bands were observed at 3459, 2617, 1638, 1389,1276, 1016 and 624.7 for SeNPs and 3446, 2920, 2853, 1639 and 546.9 for Ch-SeNPs (Fig. 8). The presence of nanoscale particles of selenium biosynthesized by fungal filtrate was detected in SEM connected with EDX analysis as regular well dispersed circular particles in the image as shown in Fig. 9a. The spectrum of the energy dispersive spectroscopic analysis (EDX) confirmed the presence of SeNPs as shown in Fig. 9b. This analysis is carried out to show the amount of selenium nanoparticles in the sample. The spectrum appeared in the figure showed the presence of SeNPs and the presence of other elements in the solution mixture such as Ca (21.38%), Mg (2.77%), C (21.83%), O (60.12), and Na (9.03), Al (1.68), and Se (0.24). The concentration of selenium nanoparticles solution was estimated by ICP analysis. The recorded results showed that the nanoparticles solution, prepared from 3 mM SeO_2,_ had 125.34 ± 6.34 mg^−l^ of selenium.

#### Efficiency of SeNPs and Ch-SeNPs on larva of *S. littoralis*

Different concentrations of the biosynthesized nanoparticles (50, 100, 150, 200 ppm) were examined against the larva of cotton worm. The results showed that 200 ppm gave the highest rate of larval mortality. Table 3 revealed that the percentage of larval mortality reached its maximum value after 10 days of treatment with 200 ppm of SeNPs and Ch-SeNPs (80 and 88%, respectively). It was observed that Ch-SeNPs gave higher mortality rate than SeNPs.

**Table 3:** Effect of Ch-SeNPs and SeNPs biosynthesized by *P. griseofulvum* against larval mortality.

LSD_0.05_	F test	*P*	15 day	10 day	7 day	3 day	1 day	Treatment
1.6272	***	0.0001	88a	88a	75b	57c	34d	Ch-SeNPs
1.6272	***	0.0001	80a	80a	70b	50c	30d	SeNPs
1.6272	***	0.0001	0%	0%	0%	0%	0%	Control

The mean value followed by different letters (a, b, c, d &e) within the same row are significantly different (One-way ANOVA, *P* ≤ *0.05*).

LSD: the least significant difference.

*** means highly significant.

#### Efficiency of spore suspension, SeNPs and Ch-SeNPs on larva of *P. griseofulvum* against larval and pupal stages of *S. littoralis*.

The inhibition effect of spore suspension of *P. griseofulvum*, Ch-SeNps, SeNPs and were evaluated against Larval duration, larval mortality, pupal period, and pupation, pupal mortality and adult emergence. The optimum concentration of spore suspension producing maximum inhibition against larval and pupation stages of *S. littoralis* were 9.8*10^12^ (Table 4). The recorded results detected that the Ch-SeNPs have the highest mortality effect against larval and pupal stages than SeNPs and spore suspension (Table 5).

**Table 4:** Screening studies for the effect of spore suspension of *P. griseofulvum* against larval and pupal stages.

Treatments	Larval duration/day	Larval mortality/%	Pupal period/ day	Pupation%	Pupal mortality %	Adult emergence
9.8*10^12^	12a	72.33a	6b	25c	9.33c	15.67cd
9.8*10^11^	6c	67b	6.33b	31d	13.76b	17.24c
9.8*10^10^	5c	61.66c	5b	35.33c	20a	15.33d
9.8*10^9^	6.67c	52.33d	6.67b	44.67b	19a	25.67b
Control	8.67b	0.00e	6.67b	95.33a	4d	91.33a
F test	***	***	*	***	***	***
LSD.05	1.695	1.724	1.819	1.819	18.19	1.831

The mean value followed by different letters (a, b, c, d, e) within the same column are significantly different (One-way ANOVA, *P* ≤ 0.05).

LSD: the least significant difference.

*** means highly significant.

**Table 5:** Screening studies for the Effect of Ch-SeNPs (200 ppm) and spore suspension (SP) of *P. griseofulvum* against larval and pupal stages.

Treatments	Larval duration/day	Larval mortality/%	Pupal period/day	Pupation %	Pupal mortality %	Adult emergence
Ch-SeNPs	15a	88a	8.6a	10d	9.67a	0.33d
SeNPs	14a	80b	7.2ab	15c	11.2a	3.8c
SP (9.8*10^12^)	12b	72.33c	6b	25b	9.33a	15.67b
Control	8.6c	0.00d	6.66b	95.33a	4b	91.33a
*P*	0.0002	0.0001	0.0631	0.0001	0.0001	0.0001
F test	***	***	NS	***	***	***
LSD _0.05_	1.883	1.631	1.883	1.883	1.883	1.635

The mean value followed by different letters (a, b, c, d, e) within the same column are significantly different (One-way ANOVA, *P* ≤ 0.05).

LSD: the least significant difference.

*** means highly significant.

NS means noun significant.

#### Toxicological effect of SeNPs and Ch-SeNPs on* S. littoralis*

As shown in Table 6, the recorded results detected that the Ch-SeNPs were the highest toxic compound against the 2nd instar larvae. The values of LC50 of larvae treated with SeNPs and Ch-SeNPs were 112.103 and 88.199, respectively and these values were 438.703, and 322.137 at LC90 after 24 h. of treatment. Regarding toxicity index, the results indicated that SeNPs recorded the lowest toxicity index at LC50 (78.68) with slope values of (1.6787). The toxicity of Ch-SeNPs was 1.27 and 1.36 times, at LC50 and LC90, respectively, over the toxicity of SeNPs.

**Table 6:** Toxicity effect of SeNPs and Ch-SeNPs by *P. griseofulvm* on the treated *larvae.*

Treatments	LC_50_ (ppm)	Confidence limits		LC_90_ (ppm)	Confidence limits		Slope	Toxicity index		Relative potency	
		Lower	Upper		Lower	Upper		LC_50_	LC_90_	LC_50_	LC_90_
SeNPs	112.103	93.269	134.703	438.517	300.833	911.865	1.6787	78.68	73.46	1.00	1.00
Ch-SeNps	88.199	71.065	104.157	322.137	238.499	558.479	1.768	100	100	1.27	1.36

#### Biochemical activity of selenium nanoparticles against 2nd larva of *S. littoralis*

The biochemical activity of *S. littoralis* larvae, such as carbohydrates enzymes, total soluble protein, and acetylcholine esterase, was evaluated after 24 h of treatments with SeNPs and Ch-SeNPs (Table 7). The results revealed that the *S. littoralis* treated with SeNPs disrupted the activity of invertase, amylase, and trehalas enzymes by increasing or decreasing, compared to the untreated samples. It was observed that the enzyme activity of *S. littoralis* larvae, treated with nanoparticle solutions, was disordered. The Ch-SeNPs high significantly increased the amylase activity (288.04 µg^−g^ glucose) over the control (156.63 µg^−g^ glucose). The treatment with nano-selenium solutions recorded a highly significant decrease in the activity of the invertase enzyme. The highest effect in invertase activity was observed in the samples treated with Ch-SeNPs, where the enzyme activity decreased to114.38 µg^−g^ glucose compared to the untreated sample (487.32 µg^−^g glucose). It was also noticed that nano-selenium solutions caused an increase in the activity of the trehalase enzyme. The Ch-SeNPs increased their activity to 2594.96 µg^−g^ glucose compared to control (1667.16 µg^−^g glucose). A non-significant effect was observed in the total soluble protein of *S. littoralis* larvae at all treatments. All treatments caused decreasing in the activity of acetylcholine esterase (AChE). The highest decrease was observed in the case of treatment with Ch-SeNPs (21.8 U^−^g) compared to control (71.87 U^−g^).

**Table 7:** Effect of SeNPs and Ch-SeNPs synthetized by fungal filtrate of *P. griseofulvum* on some physiological activities of the cotton leafworm *Spodoptera littoralis.*

Producer strain	Treatments	Acetyl choline esterase (U^−g^)	Total soluble protein (mg^−g^)	Carbohydrate enzymes (µg glucose^−g^)		
				Amylase	Invertase	Trehalase
*P. griseofulvum*	Filtrate	61.20b	7.464ab	105.26d	336.84b	1635.99 c
	SeNPs	45.01c	7.139ab	257.75b	289.78c	2064.73 b
	Ch-SeNPs	21.8d	7.76a	288.04a	114.38a	2594.96 a
Control	71.87a	6.78b	156.63c	487.32a	1667.16 c	
*P* value	0.0001	0.0559	0.0001	0.0001	0.0001	
F- test	***	NS	***	***	***	
LSD_0.05_	2.9509	0.6999	12.2243	31.7276	235.0016	

The small letters in the column with the same symbols mean values with non-significant difference.

The small letters in the column with different symbols mean values with significant difference.

SD: means triplicate measurements of two independent experiments. NS means noun significant.

#### Transmission electron microscope (TEM) analysis of treated larva of *S. littoralis*

**Fig. 10:**
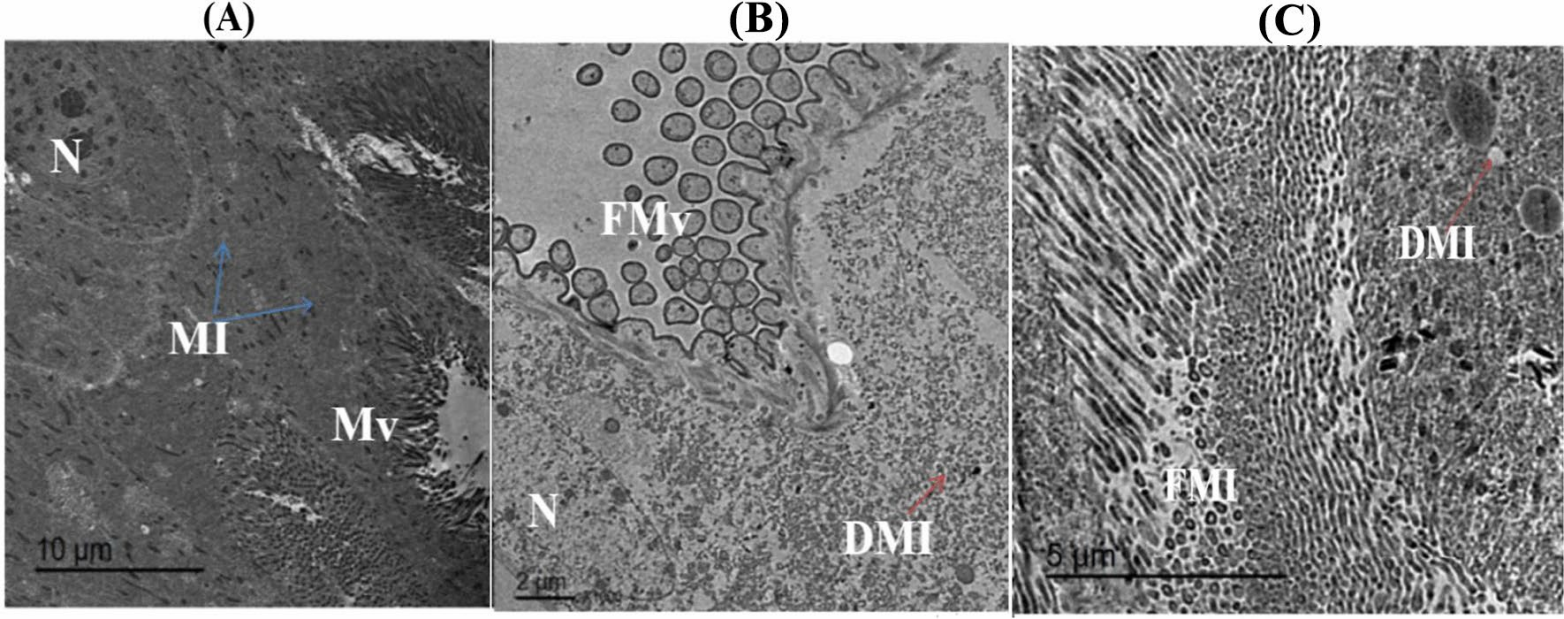
Photomicrograph TEM illustrating microvilli region in midgut of *S. littoralis* 2nd instar larvae befor (**A**) and after treatment with Ch-SeNPs (**B** and **C**). Mi: mitochondria, Mv: microvilli, N: nucleus DMi: damedged mitochondria,FMv: fragmented microvilli, N: nucleus.

The image of TEM analysis showed that the midgut of untreated larva (control) was characterized by normal microvilli, well- develop nucleus, and rich with mitochondria (Fig. 10a). The ultrastructure of midgut tissue of untreated *S. littoralis* larvae showed normal epithelial cells with clear cytoplasm and tall basophilic nuclei, loose networks of rough endoplasmic reticulum while the lining of the midgut cavity had well defined organized microvilli looked like a brush in a parallel line on the outer boundary. On the other hand, the TEM image (Fig. 10B, C) appeared fragmented microvilli and damaged mitochondria and nucleus in microvilli region of the midgut of treated 2nd instar larva of *S. littoralis*. The ultrastructure analysis, appeared in Fig. 10B, showed deterioration in the midgut tissues of *S. littoralis* larvae treated with Ch-SeNPS as well extreme dilation in endoplasmic reticulum. The microvilli appeared vacuole-like configuration, withered and shedding. Additionally, many large vacuoles appeared in the cytoplasm beside in some section ultrastructure deformities in the cytoplasm. It was also observed that the cytoplasm electron was semitransparent and lacking organelles. The epithelial cell showed alterations commonly observed in Fig. 10C including irregular structure of epithelium, destroyed microvilli with different degrees of deformations, swallowing, clumped, and shrunk. Considerable swelling in the mitochondria and partial to complete loss of mitochondrial cristae was also detected in addition to the appearance of numerous phagolysosomes.

#### Cytotoxic activity of selenium nanoparticles

The cytotoxic activity of selenium nanoparticles was investigated by their ingestion with honeybee foragers. The obtained results showed that all bio-synthesized SeNPs and Ch-SeNPs were safe to honeybee workers in general. The percentage of mortality from exposed workers did not exceed 1% in stock solutions.

#### Transmission electron microscopic analysis of treated honeybee

**Fig. 11:**
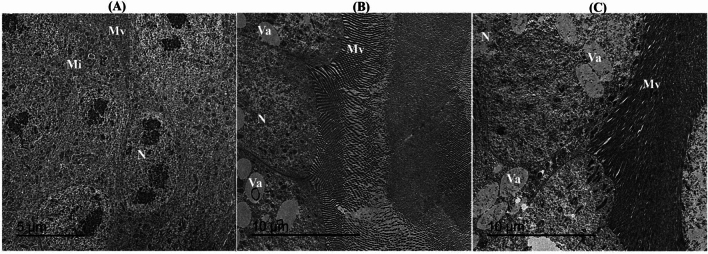
Photomicrograph of TEM illustrating microvilli region in mid gut of untreated (**A**) and Ch-SeNPs treated honey bee (**B** and **C**). Mv: microvilli, N: nucleus, Va: vacuoles.

Ultrastructure analysis of honeybee mid gut tissues of the treated groups and the control was conducted to explore the difference regarding organelles structure. Considering the control group, it was obvious that the mid gut epithelial cell emerged as a line with brush border microvilli, well defined nuclei, distinct and well chromatin networks with defined nuclear membrane, and granular cytoplasm with dispersed mitochondria (Fig. 11A). Additionally, arc-like mitochondria, swollen lysosomes, and enlarged phagosomes were observed in midguts of worker bees fed by Ch-SeNPs. Nanoparticles were not observed in the cell nuclei and in the other organelles, but relatively large vesicles and phagolysosomes are diagnostic in the cytoplasm (Fig. 11B). Slight characteristic ultrastructure changes in the midgut of honeybees were detected after feeding a treated diet (Fig. 11C). Also, nucleus has fragmented nucleoli and no clumped chromatin while phagolysosomes are increased in numbers and lysosomes that enclosing nanoparticles.

## Discussion

Decades ago, artificial insecticides were employed to manage insect pests. These chemical pesticides lead to various health issues for all living organisms^23^. Recently, there has been significant progress in utilizing nanoparticles to enhance pest control in plant protection management^24^. Hence, the present research endeavors to decrease reliance on chemical pesticides, and advocate for the adoption of safer alternatives such as nanoparticles for pest management. Selenium is regarded as the most secure, costless, and affordable metal. Selenium nanoparticles are an acceptable option to be selected as a novel pest control approach in our study^25^.

Between 25 fungal strains, *P. griseofulvum* was selected as the most potent fungal strain for biosynthesis of SeNPs. The biosynthesis of SeNPs by fungal filtrate is a favorable approach because of their higher biomass, ease of cultivation, tolerance to different concentrations of metals, and potential bioaccumulation of metals compared to bacteria^26^. *P. griseofulvum* was used for synthesis of both SeNPs and Ch-SeNPs. The initial indication of selenium nanoparticle formation, using fungal filtrate, was the transition in the solution’s hue, shifting from a transparent colorless to a yellowish-red shade. This alteration in color arises from the stimulation of unbound electrons, creating absorption bands through surface plasmon resonance (SPR) due to the synchronized vibration of electrons in resonance with light waves^27,28^. The production of SeNPs was verified through UV–visible spectroscopic analysis^25,29,30^. The UV–visible spectra exhibited peak absorbances between 260 and 280 nm, attributed to the stimulation of electrons in the conductive band near the surface of the nanoparticles^31^. Also, Arunthirumeni et al.^32^ showed the presence of a maximum peak at 259 nm due to the formation of SeNPs. The mechanism by which the SeNPs could be synthesized by *P. griseofulvum* extract may be explained by the large amounts of bioactive molecules, reducing enzymes, and extracellular proteins produced by fungi. These biomolecules have intense reducibility and act as reducing and capping agents involved in the extracellular biosynthesis of nanoparticles. They can reduce the tetravalent selenium ions to selenium. Additionally, amino and carbonyl groups in fungal biomacromolecules have strong complex effects on selenium and selenium ions. They can be enveloped on the surface of nano-selenium and play a significant role in the protection and dispersion of SeNPs. FTIR analysis of SeNPs and Ch-SeNPs showed strong observed bands at 2617 and 2853 cm^−1^. The appearance of these peaks indicated the presence of carboxylic acid as a functional group in the solution mixture. Abd-Elraoof et al.^33^ detected strong bands at 2921 cm^−1^ and 2872 cm^−1^ which are typical polysaccharide bands. The bands that appeared at 1638 and 1639 cm^−1^, indicated the presence of –NH and diketones group. Joshi et al.^34^ found major absorption bands at 1638.83 cm^−1^ in SeNPs solution which corresponds to the presence of –NH groups in the nanoparticle’s solution. The bands appeared at 624.7 and 546.6 indicated the presences of halogen compound (Chloro compound) (C–Cl) in the solution mixture. The presence of proteins in the nanoparticle solution was indicated by C–N and C–C stretching at 1634–1654 cm^−1^^33,35^. The presence of these functional groups in nanoparticle solution through FTIR analysis confirms the presence of different active biomolecules, such as protein, enzyme, and polysaccharides which may have a principal role in the synthesis of nanoparticles^36,37^. Similar to the current results, strong peaks were found in SeNPs and Ch-SeNPs solution at 570, 1090, 1634, 2361, and 3320 cm^−1^, which respectively corresponded to haloalkanes, C–O stretching, C–N and C–C stretching, O=C=O bending, and N–H and O–H stretching^10^.

Examination through TEM revealed the presence of polygonal and spherical particles in SeNPs produced by fungal filtrates, shifting towards more regular shapes upon chitosan-coating nanoparticles. Both TEM and DLS assessments demonstrated that *P. griseofulvum* OR672743 yielded particles of small size. The same feature was previously investigated with copper nanoparticles and gelatine-coated nanoparticles produced by *A. wentti* filtrate^38^. The SEM–EDX analysis of SeNPs and Ch-SeNPs was carried out to investigate their elemental content and morphology. The SEM–EDX image demonstrated the spherical shape of nanoparticles and the presence of selenium element. The spectrum peaks of EDX analysis confirm the presence of selenium in the nanoparticle solution. This result agrees with SeNPs biosynthesized by *Trichoderma atroviride* and *Streptomyces parvulus*^10,34^. Signals of calcium, magnesium, and sodium can also be seen in the figure. The presence of these salts in the mixture is due to the media components used for culture growth and the metabolic products of *P. griseofulvum*. Strong signals of oxygen and carbon can be observed in the spectrum. These elements may be due to the proteins present along with nanoparticles and the organic substances that act as capping agents on the surface of the selenium nanoparticle. Additionally, ICP analysis detected the presence of selenium in the nanoparticle’s solution. All obtained results confirmed the biosynthesis of SeNPs and Ch-SeNPs successfully in our lab. The bio fabrication of selenium nanoparticles results from the microbial production of reductases which can reduce oxy-anion SeO42 (water-soluble compound) to SeO32, then to selenium Se0 (insoluble element). Fungal biosynthesis of SeNPs attracts attention due to the production of different extracellular products in the fungal metabolite that plays an important role in nanoparticle formation and stability. Different compounds such as terpenoids, vitamins, proteins, tannins, polysaccharides, flavonoids, and other biomolecules may be responsible for the reduction and stabilization process, and capping of nanoparticles, preventing their aggregation in the aqueous solution during the biosynthesis process^39^. Fouda et al.^40^ reported that the biosynthesis of extracellular SeNPs by *Penicillium crustosum* is owing to the possibility of the presence of various enzymes such as amylase, cellulase, gelatinase, and xylanase. The physicochemical characteristics of nanoparticles are highly dependent on their size and morphology. To produce an effective size distribution and high production of nanoparticles, it is necessary to optimize the physicochemical parameters such as culture medium on which the microorganism was grown, and the reaction conditions including temperature, reaction time, and pH value^41^. Our optimization results indicated that the reaction mixture with pH 9 was the optimum for biosynthesis of SeNPs from *P. griseofulvum*. The incubation temperature and different periods of incubation are principal factors in any chemical and biological reaction for nanoparticles production and they affect the rate of reaction^42^. The present results showed that the incubation of the solution mixture at 40 °C for 7 days was the optimum condition for biosynthesis of SeO2NPs by *P. griseofulvum*, where the maximum absorbance intensity was detected at these conditions. It was noticed from this experiment that the increment in incubation temperature rapid the formation of nanoparticles, where the nanoparticles were synthesized after only 24 h of incubation at 60 °C. This condition is not suitable for activation of reductase enzymes, the thing indicates the presence of additional biomolecules in fungal filtrates which may be responsible for nanoparticles biosynthesis. Sarsar et al.^43^ showed that the reduction of silver ions to AgNPs depended on temperature, and this could be associated with the enzyme’s stability existing in the fungal extract. The highest yield of nanoparticles was observed at 40 and 50 °C during shorter incubation periods. Similarly, the nanoparticles were synthesized at pH levels of 7.0, 8.0, and 9.0, with the greatest production at pH 8.0 and 9.0 at lower incubation periods^44^. This outcome suggests that the biosynthetic source predominantly influences the formation of nanoparticles. Those particles coated with chitosan displayed superior dispersion and were smaller in size compared to the uncoated particles. Chitosan can be used as a protective antioxidant coating agent where it is a biodegradable natural polymer derived from the exoskeleton of crustaceans^45^. Because of its biocompatibility and non-biotoxicity, chitosan is widely used in the fields of medicine and agriculture. This is primarily due to its inherent antibacterial properties, which can be harnessed both independently and in combination with various metal ions. Therefore, chitosan served as a stabilizing and protective agent, facilitating the dispersion of particles and inhibiting their clumping. As a result, the coated particles exhibited a smaller size compared to their uncoated counterparts. This explanation was confirmed by Zeta potential analysis which detected higher charge on the surface of Ch-SeNPs than SeNPs. Chitosan is regarded as a secure, naturally derived substance suitable for enveloping SeNPs. This encapsulation aids in preserving their integrity by averting aggregation and thwarting oxidation. Consequently, the nanoscale properties remain unaltered for an extended duration, ensuring their longevity^33^. Biosynthesized SeNPs and Se/Ch-nanoconjugate were convincingly recommended for biomedical applications as potent antimicrobial, versatile, and anticancer agents, ensuring efficacy, notable biosafety, and environmental compatibility^10^. Chitosan nanoconjugate tends to stabilize the substrate materials to prevent side-interactions with environmental factors and improve the surface properties such as conductivity, magnetic, adsorption, and optical perfectness^43,46^.

In this study, both SeNPs and Ch-SeNPs were tested against all growing stages of cotton leafworm. The results demonstrated that the treatment of 2nd instar larva of *S. littoralis* with biosynthesized SeNPs significantly increased the percentage of larval mortality. El-Helaly et al.^47^ agree with our investigation, who found that nano-silica caused 89.82–95.00% larval mortality of *S. littoralis*. El-Ashmouny et al.^48^ proved that the green silver nanoparticles have significant toxicity against the 4th instar larvae of the cotton leafworm. They reported that the prepared nano-silver significantly impacted Spodoptera antioxidants, leading to oxidative stress and cell death. In this study, it was noted that larvae treated with Ch-SeNPs exhibited elevated mortality rates compared to those treated with SeNPs. Ch-SeNPs demonstrated the highest mortality percentage and the shortest larval stage duration. Selenium nanoparticles induce the production of Reactive Oxygen Species (ROS) in the pest cells. Its elevation can lead to oxidative damage to cellular components, including lipids, proteins, and DNA. Moreover, the accumulation of ROS in *S. littoralis* may lead to oxidative stress, which disrupts cellular homeostasis. On the other hand, SeNPs can impair the pest’s antioxidant defense systems by inhibiting enzymes like catalase and superoxide dismutase, further exacerbating oxidative stress. This results in mitochondrial dysfunction and reduces energy production leading to cell death. Conjugation of chitosan with SeNPs increases the positive charge on Ch-SeNPs resulting in vital cellular uptake of nanoparticles, enhancing the membrane permeating abilities of the living cells, and thus increasing the apoptosis-inducing activities^49,50^. Additionally, the application of all bio-synthesized SeNPs directly led to a remarkably substantial decrease in pupation, as well as reductions in pupal duration for *S. littoralis*. Generally, Ch-SeNPs severely reduced the percentage of pupation compared to control. A similar result was recorded by Abd El-Latef et al.^51^, who observed that the treatment of *S. littoralis* with nano-copper and nano-zinc particles reduced its pupation and all other biological aspects, announced a reduction in feeding consumption after their treatment with nanoparticles. Additionally, it was discovered that both coated and uncoated nanoparticles, when applied directly, exhibited the most pronounced lethal impact on the larval and pupal phases of *S. littoralis*. The significantly potent influence of these nanoparticles on both larval and adult stages of the cotton leafworm may be attributed to their ability to infiltrate causing damage to the insect exoskeleton. These effects may be owing to their substantial surface area relative to their bulk counterparts^38^. Through this process, the protective water barrier on the insect’s cuticle is disrupted, causing the insect to gradually lose water and eventually perish. Furthermore, the nanoparticles produced from fungal filtrate through biosynthesis lowered the population density of the insects by impacting their biological characteristics. This outcome is likely a consequence of the nanoparticles infiltrating the lipid layers of the insect’s cuticle, resulting in harm to the protective wax coat and ultimately leading to demise through dehydration^52,53^. The detrimental impact of nanoparticles on *S. littoralis* may also stem from their capacity to breach cell membranes, gaining access to mitochondria and ultimately triggering harmful reactions^54^. Sahayaraj et al.^55^ reported that nanoparticles were swiftly consumed by insects, entering the hemolymph via the insect gut lumen and subsequently appearing in the frass. These nanomaterials have the potential to hinder larval nutrition by directly influencing the chemo sensilla of the larvae, thereby disrupting their feeding behavior. Some histopathological changes appeared in the midgut tissues. They were represented as destroying the integrity of the epithelial cells and the ciliated border. The columnar cells began to disintegrate, and the peritrophic membrane became vacuolized. In contrast, the cuticle layers were not affected by various treatments^56^.The mod of action of nanoparticles on the leaf worm insect was proved by histopathological analysis of the gut region of treated larvae which demonstrated elongation of epithelial cells. The results ensured the destructive effect and structural alterations in different tissues of larvae treated with SeNPs and Ch-SeNPs. The images of SEM showed the appearance of vacuoles, the absence of a brush border, and empty goblet cells. Genetic materials could not be detected within the nucleus. Destruction of the musculature of the gut was also detected^48^. The damage of some organelles in the midgut of *S. littoralis* after treatment with SeNPs may be due to its ability to penetrate cell membranes, enter mitochondria, and eventually excite injurious reactions. The explanation of the mode of action of nanoparticles against insects is still a topic of discussion, and many hypotheses have been made by various researchers. Cytotoxicity experiments on honeybees are critical for safeguarding not only these beneficial insects but also the broader environment, human health, and sustainable agricultural practices. It is an essential component of responsible stewardship in agriculture and environmental management. SeNPs recorded safe effect on honeybees when applied as nutrition with sugar syrup. Honeybees are key agricultural pollinators and plant pollen. They feed on the nectars of flowers which contain many phytochemicals that up-regulate the expression of detoxification genes and can increase bees’ tolerance to some pesticides and other toxic materials^57,58^. Our finding is similar to Ammar and Abd-ElAzeem^38^, who investigated that all biosynthesized cupper nanoparticles were safe to honeybee workers in general when incorporated in sucrose syrup and the percentage of mortality in exposed workers did not exceed 1% in stock solution. Colonization of honeybee digestive tracts by *L. thermotolerant* revealed that this yeast species maintains high levels in the honeybee midgut only at temperatures below the typical colony temperature^59^. Although many fungal species are found in association with honeybees and their broader environment, the effects of these interactions on honeybee health are largely unknown. They showed that the yeast’s ability to maintain high levels in the digestive tract is influenced by temperature and can lead to alterations of the microbiome in young bees^59^. The activities of key antioxidant enzymes protect the bee’s organisms against free radicals and thus delay the aging processes^60^. Nanoparticles have become a focus in pest control because of their unique properties that can help target pests while minimizing harm to beneficial organisms like honeybees.

## Conclusion

It is concluded from this study that SeNPs and Ch-SeNPs can be easily synthesized by an economic, costless route using fungal filtrates of *P. griseofulvum*. These nanoparticles had highly significant anti-insect activity against the *S. littoralis*. The selenium chitosan nanocomposite had a regular spherical shape and was smaller than SeNPs at all experimental conditions. Additionally, both SeNPs and Ch-SeNPs were more influential for the inhibition of cotton worms than the fungal spore suspension. The anti-insect activity of the biosynthesized nanoparticles depended greatly on their size and stability of nanoparticles. Ch-SeNPs were more stable than SeNPs where they have a higher positive charge on their surface. The bio-fabricated SeNPs and Ch-SeNPs, using a safe ecofriendly method is a good method and new procedure that can be used as bio-control agent against the cotton leafworm *S. littoralis.* This study is the first report about biosynthesis of SeNPs and Ch-CuNPs by *P. griseofulvum* and their application as biocontrol agent against *S. littoralis*.

## Data Availability

The datasets used and analyzed within the current study are available from the NCBI website. The ITS rRNA sequence of *P.* *griseofulvum* was deposited to NCBI GenBank under the accession number OR672743 (https://www.ncbi.nlm.nih.gov/nuccore/OR672743.1).
